# Huaier Polysaccharide Interrupts PRV Infection via Reducing Virus Adsorption and Entry

**DOI:** 10.3390/v14040745

**Published:** 2022-04-01

**Authors:** Changchao Huan, Jingting Yao, Weiyin Xu, Wei Zhang, Ziyan Zhou, Haochun Pan, Song Gao

**Affiliations:** 1 Institutes of Agricultural Science and Technology Development, College of Veterinary Medicine, Yangzhou University, Yangzhou 225009, China; changchaohuan@yzu.edu.cn (C.H.); yaojingting201609@163.com (J.Y.); xuxuwy@163.com (W.X.); weizhang0918@163.com (W.Z.); yzdxzzy@163.com (Z.Z.); haochunpan0826@163.com (H.P.); 2 Jiangsu Co-Innovation Center for Prevention and Control of Important Animal Infectious Diseases and Zoonoses, Yangzhou 225009, China; 3 Key Laboratory of Avian Bioproduct Development, Ministry of Agriculture and Rural Affairs, Yangzhou 225009, China

**Keywords:** Huaier polysaccharide, pseudorabies virus, antiviral, infection

## Abstract

A pseudorabies virus (PRV) novel virulent variant outbreak occurred in China in 2011. However, little is known about PRV prevention and treatment. Huaier polysaccharide has been used to treat some solid cancers, although its antiviral activity has not been reported. Our study confirmed that the polysaccharide can effectively inhibit infection of PRV XJ5 in PK15 cells. It acted in a dose-dependent manner when blocking virus adsorption and entry into PK15 cells. Moreover, it suppressed PRV replication in PK15 cells. In addition, the results suggest that Huaier polysaccharide plays a role in treating PRV XJ5 infection by directly inactivating PRV XJ5. In conclusion, Huaier polysaccharide might be a novel therapeutic agent for preventing and controlling PRV infection.

## 1. Introduction

Pseudorabies virus (PRV) has been regarded as one of the major causative agents for fatal losses in the swine industry worldwide [1,2]. PRV is a double-stranded linear DNA and enveloped virus that belongs to the subfamily alphaherpesvirinae of the family Herpesviridae [3,4]. PRV can infect various species of mammals, such as swine, wild boars [5], ruminants (e.g., goat, sheep, and cattle) [6,7], carnivores (e.g., hunting dogs, minks, and foxes) [8,9], and rodents. Swine are the unique natural host and reservoir of PRV [10]. PRV infections in swine are often fatal, and the infected swine die from central nervous system disorders and respiratory diseases [3]. Pregnant sows infected with the virus exhibit abortions, stillbirths, and mummified fetuses and are often infertile, with a high rate of rebelliousness [11]. The Bartha-K61 strain is still one of the most widely used live virus vaccines to confront PRV infections worldwide [4]. Pseudorabies (PR) incidence was first reported among cats in 1947 in China; thereafter, PR incidence was reported in other species, particularly in the pig industry [12]. Before 2011, the Bartha-K61 vaccine could prevent and control PR in China; however, the emergence of several variant strains led to the new pseudorabies epidemic among immunized swine [13]. PRV variants exhibit high sequence divergence compared with classical PRV strains [14]. The Chinese triple-gene-deleted (gE/gI/TK) vaccine and TK/gG-deleted vaccine have been extensively used to prevent PR in the Chinese pig population. However, the protection efficacy of the two vaccines has not been reported [3]. More importantly, PRV could infect humans, and it was isolated from an acute human encephalitis case [15,16,17]. Therefore, identifying new preventive and therapeutic measures for controlling PR, in addition to vaccines, is imperative.

The application of traditional Chinese medicine to treat diseases can be traced back to 200 Anno Domini (AD) [18]. Growing evidence suggests that the traditional Chinese medicine offers numerous compounds having antiviral activities [19]. Trametes robiniophila murr (Huaier), as a traditional Chinese medicine, has been used to treat many diseases for more than one thousand years [20]. Huaier extract can regulate DNA-dependent transcription and the cellular response to hypoxia during breast cancer development and progression [21]; it can also enhance the host immunity and induce apoptosis in breast cancer cells [22]. In addition, studies have confirmed that Huaier granule exhibits good safety in clinical antitumor therapies [23]. In particular, it was considered as a promising adjuvant for the treatment of breast cancer when used in combination with conventional treatment [24]. Huaier polysaccharide also exhibits excellent safety, therapeutic efficacy, and minimal side effects in the clinical treatment of lung cancer, liver cancer, and other solid tumors [25]. However, the effect of Huaier polysaccharide on PRV infection has not been investigated. In this study, we evaluated the mechanism of Huaier polysaccharide in PRV infection; our findings suggested that Huaier polysaccharide inhibits PRV XJ5 adsorption, entry, and replication and thus can dampen PRV infection.

## 2. Materials and Methods

### 2.1. Cells and Viruses

Pig kidney (PK15) cells and African green monkey kidney (Vero) cells were stored at the Yangzhou University Infectious Diseases Laboratory and grown in monolayers at 37 °C under 5% CO_2_ conditions. PK15 cells and Vero cells were cultured in Dulbecco’s modified Eagle medium (DMEM) supplemented with 5% and 6% fetal bovine serum (FBS; Lonsera, Uruguay), 100 units/mL penicillin, 100 μg/mL streptomycin sulfate, and fungizone.

PRV XJ5, PRV NT, and PRV Ra were stored at the Yangzhou University Infectious Diseases Laboratory. We used 0.1 multiplicity of infection (MOI) of PRV-infected PK15 cells in all experiments.

### 2.2. Reagents and Antibodies

In all subsequent experiments, Huaier polysaccharide (Yangling Ciyuan Biotechnology Co., Ltd., Xian, China) was diluted with PBS to prepare stock solutions of 50 mg/mL and stored at −20 °C. The antibodies for PRV gB were generated by immunization of mice with purified recombinant gB in our laboratory. Actin Ab—T0022 was procured from Affinity Bioscience (Beijing, China). FITC-conjugated goat anti-pig IgG antibody combining PRV-positive sera was purchased from Sigma-Aldrich (St. Louis, MO, USA). DAPI (#C1006) was purchased from Beyotime Biotechnology (Shanghai, China).

### 2.3. Cell-Based Infectivity Assays

#### 2.3.1. Cells Viability Assay

Cell viability was evaluated with Enhanced Cell Counting Kit-8 (CCK-8) assays, according to the manufacturer’s instructions (Beyotime Biotechnology, Shanghai, China). For cell viability assays, cells were seeded at 5000 per well into 96-well plates. On the next day, the medium was changed to DMEM/5% FBS supplemented with different concentrations of Huaier polysaccharide for 24 and 36 h. CCK-8 (10 μL) was then added to each well, and the cells were incubated for 1 h at 37 °C. The absorbance was detected at 450 nm with a microplate reader (Bio-rad, Hercules, CA, USA).

#### 2.3.2. General Effect of Huaier Polysaccharide on PRV Infection

Huaier polysaccharide was added at 37 °C for 24 h to verify its effect on PRV infection. DMEM containing 5% FBS was used to grow the PK15 cells in a 37 °C/5% CO_2_ incubator. The PK15 cells were seeded in a 6-well plate, with a density of 5 × 10^5^ cells per well. When the cells grew to approximately 8.5 × 10^5^, the PK15 cells were infected with PRV XJ5 (MOI = 0.1) for 1 h. At 1 h post infection (h.p.i.), DMEM was removed, and the cells were washed with PBS. The cells were incubated with different concentrations (25, 50, 100, or 200 μg/mL) of Huaier polysaccharide in 2% FBS DMEM (2% DMEM). On the next day, the cells were collected to determine PRV XJ5 protein expression through Western blotting and indirect Immunofluorescent Assay (IFA), intracellular viral DNA copies were analyzed by qRT-PCR after 24 h at 37 °C, and cell supernatants were used to measure the virus titer through TCID_50_ assay.

#### 2.3.3. Exploration of Huaier Polysaccharide’s Effect on PRV Adsorption and Entry

Huaier polysaccharide was added at 4 °C for 1 h and at 37 °C for 1 h to verify its effect on PRV adsorption and entry. The PK15 cells were cultured using 1 mL of DMEM without FBS to dilute Huaier polysaccharide to 25, 50, 100, 200 μg/mL and then infected with PRV XJ5 (MOI = 0.1) at 4 °C for 1 h. Thereafter, the cells were washed thrice with cold PBS and incubated in 2% DMEM containing appropriate concentrations of Huaier polysaccharide for 1 h at 37 °C. Then, cells were washed with citric acid to remove extracellular virus and again washed with PBS. Adding 2 mL 2% DMEM, intracellular viral proteins were detected through Western blotting and IFA, and the contained viruses’ cell supernatants were used to measure virus titers by using the TCID_50_ assay at 24 h.p.i. Intracellular viral DNA copies were analyzed by qRT-PCR after 1 h at 37 °C. 

#### 2.3.4. General Effect of Huaier Polysaccharide on PRV Adsorption

Huaier polysaccharide was added at 4 °C for 1 h to verify its effect on PRV adsorption. The PK15 cells were digested with 0.25% trypsin and diluted with DMEM containing 5% FBS, dripped onto a 6-well plate at a concentration of 5 × 10^5^ cells/well in a 37 °C/5% CO_2_ incubator. When the cells grew to approximately 70–80%, the cells were treated with different concentrations of Huaier polysaccharide and infected with PRV XJ5 (MOI = 0.1) at 4 °C for 1 h. The cells were cleaned thrice with cold PBS and maintained in 2% DMEM for 24 h. Intracellular viral proteins were detected through Western blotting and IFA. Cell supernatants were used to measure virus titers through TCID_50_ assay. Viral DNA copies of adsorption were analyzed by qRT-PCR after 1 h at 4 °C.

#### 2.3.5. Exploration of Huaier Polysaccharide’s Effect on PRV Entry

Huaier polysaccharide was added at 37 °C for 1 h to verify its effect on PRV entry. The PK15 cells were infected with PRV XJ5 (MOI = 0.1) at 4 °C for 1 h without Huaier polysaccharide. The infected cells were then washed with cold PBS three times and cultured in 2% DMEM containing different concentrations of Huaier polysaccharide at 37 °C for 1 h. The cells were washed thrice with citric acid and PBS. Then, cells were cultured in 2% DMEM without Huaier polysaccharide. Intracellular viral proteins were detected through Western blotting and IFA after 24 h at 37 °C. Cell supernatants were used to measure the virus titer through TCID_50_ assay. Intracellular viral DNA copies were analyzed by qRT-PCR after 1 h at 37 °C. The above experiments included three independent experiments.

#### 2.3.6. Effect of Huaier Polysaccharide on PRV Replication

Huaier polysaccharide was added at 37 °C for 3 h or 5 h to verify its effect on PRV replication. When the PK15 cells grew to approximately 70–80%, the cells were incubated with PRV XJ5 (MOI = 0.1) for 1 h at 37 °C and under 5% CO_2_. After the cells were washed thrice with PBS, the cells were incubated with 2% DMEM containing 25, 50, 100, and 200 μg/mL Huaier polysaccharide. At 4 h.p.i. and 6 h.p.i., the cells were collected to determine PRV-related protein expression through Western blotting and viral DNA copies were analyzed by qRT-PCR. The above experiments included three independent experiments.

#### 2.3.7. Effect of Huaier Polysaccharide on PRV XJ5

PRV XJ5 (MOI = 0.1) was pretreated with different concentrations of Huaier polysaccharide (25, 50, 100, and 200 μg/mL) for 1 h at 37 °C. Then, the cells were washed thrice with PBS. We co-incubated PRV XJ5 with cells as described above. At 24 h.p.i, the cells were collected to analyzed the expression of PRV-related proteins through Western blotting and viral DNA copies were analyzed by qRT-PCR. PRV XJ5 (MOI = 0.1) was pretreated with different concentrations of Huaier polysaccharide (100 or 200 μg/mL) for 1 h at 37 °C with 100 μL. A transmission electron microscope was used to determine the real destruction of the virions. The above experiments included three independent experiments.

#### 2.3.8. Western Blotting

Cell lysis buffer (Beyotime Biotechnology, Shanghai, China) was used to lyse the cells. The lysed cells were placed in a 1.5 mL EP tube (Eppendorf Micro Test Tubes), to which phosphorylated protease inhibitor and phenylmethanesulfonyl fluoride (PMSF) were added. The concentration of cell lysate protein was determined according to the manufacturer’s instructions for the BCA protein quantification kit. Western blotting was performed following the previously described methods [26,27]. The membranes were incubated with PRV gB or actin primary antibodies at 4 °C overnight. On the next day, the membranes were incubated with goat anti-rat secondary antibody (Beyotime Biotechnology, Shanghai, China) at room temperature for 2 h.

#### 2.3.9. Virus Titer Assays

The TCID_50_ assay was used to evaluate virus titers. After trypsinization of Vero cells, these cells were diluted with DMEM containing 6% FBS and added dropwise to a 96-well plate at a concentration of 2 × 10^3^ cells/well. Then, the cells were placed in a 37 °C/5% CO_2_ incubator until the cells adhered to the wall. After the monolayer was in the logarithmic growth phase (around 16 h), cells were washed three times with PBS, absorbing the remaining liquid. The cells were inoculated with serially diluted viruses (10^−1^–10^−7^ fold) for 1 h at 37 °C and eight replicates for each concentration were prepared. Then, 250 μL of maintenance medium was added to each well. At 72 h.p.i., we observed the CPE. Finally, CPE were counted to calculate the TCID_50_ by using the Reed–Muench method.

#### 2.3.10. Indirect Immunofluorescent Assay

After the PK15 cells were infected with PRV for 24 h, the supernatant was discarded, and the cells were washed thrice with PBS. Then, 4% paraformaldehyde that could cover the cell surface was added, and the cells were fixed at 37 °C for 15 min. Thereafter, 0.1% TritonX-100 was used to penetrate the cells for 10 min, and the cells were washed thrice with PBS. The cells were incubated with 5% BSA blocking solution at 37 °C for 2 h or 4 °C overnight. The primary antibody PRV pig-positive serum was diluted to 1:200 and incubated at 37 °C for 2 h, washed three times with PBST, and incubated with FITC-conjugated goat anti-porcine IgG antibody (Sigma-Aldrich, St. Louis, MO, USA) at 37 °C for 1 h; finally, the cells were stained with DAPI for 5 min. The cells were observed under a fluorescence microscope (LAICA, DMi8) with 488nm. All the images were captured at 100× magnification. The fluorescence density of FITC was calculated by Image J (LOCI, Madison, WI, USA).

#### 2.3.11. DNA Extraction and qRT-PCR

Briefly, we extracted DNA for each sample; 10% 92 μL SDS and 8 μL Proteinase K were added to samples and incubated at 58 °C for 1 h, followed by adding 600 μL phenol:chloroform (1:1, *v*/*v*) to each sample and vortexing, followed by centrifugation for 15 min. Then, 400 μL supernatant was collected and mixed with 800 μL anhydrous ethanol, and kept at −20 °C for 30 min. All samples were centrifuged at 4 °C for 15 min, and the precipitate was washed with anhydrous ethanol. The DNA was dissolved by ddH_2_O and incubated at 37 °C for 30 min. Primers used for qRT-PCR are as follows: gB94-F: 5′-ACAAGTTCAAGGCCCACATCTAC-3′, gB94-R: 5′-GTCCGTGAAGCGGTTCGTGAT-3′. 

#### 2.3.12. Electron Microscopy

First, we took a small amount of virus liquid or virus containing Huaier polysaccharide (100 or 200 μg/mL) droplets on the copper mesh and used filter paper to absorb excess virus solution, added 2% phosphotungstic acid dropwise on the copper mesh, used filter paper to absorb excess dye, placed the sample in a drying oven, left it to dry, and used a transmission electron microscope (Tecnai 12; Philips, Eindhoven, The Netherlands) to observe it.

#### 2.3.13. Flow Cytometry Assay

For apoptosis assays, PK15 cells were seeded at 1.2 × 10^6^ per well into 6-well plates. On the next day, the medium was changed to 2% DMEM supplemented with Huaier polysaccharide for 24 h. According to the manufacturer’s instructions, PI staining was performed with a Cell Apoptosis Kit with PI (Beyotime Biotechnology, Shanghai, China). The percentage of apoptosis cells was measured by flow cytometry on a CytoFLEX instrument (Beckman Coulter, Inc. Brea, CA, USA).

#### 2.3.14. Statistical Analysis

All the experiments were independently repeated at least three times, and all data are presented as the mean ± SD based on three independent experiments. The data were analyzed using GraphPad Prism software (GraphPad Software, SanDiego, CA, USA). One-way ANOVA, as well as Duncan’s multiple range test, was utilized to analyze differences between groups. At a *p* value of <0.05, the differences were considered to be statistically significant.

## 3. Results

### 3.1. Huaier Polysaccharide Has No Inhibitory Effect on PK15 Cells’ Growth and Apoptosis

**Figure 1:**
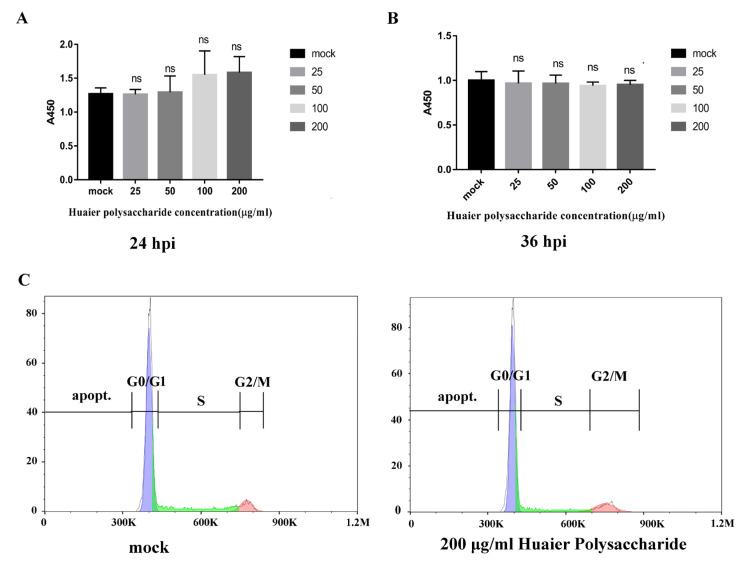
Huaier polysaccharide has no inhibitory effect on PK15 cells’ growth and apoptosis. (**A**,**B**) PK15 cells were pretreated with different concentrations of Huaier polysaccharide at 37 °C for 24 h or 36 h. Cell viability of PK15 cells was assessed with CCK-8 cell counting assays. (**C**) The apoptosis of PK15 cells treated or not with Huaier polysaccharide (200 mg/mL) was checked by flow cytometry. Significance was analyzed using the one-tailed Student’s *t*-test. Data are shown as mean ± SD based on three independent experiments. “ns” is not significant.

According to a previous study [22], results showed that the vitality of breast cancer cells is more significantly inhibited by Huaier aqueous extract. To explore the effect of Huaier polysaccharide on PK15 cells, we checked the cells’ growth using the Enhanced Cell Counting Kit-8. Figure 1A,B reveal that Huaier polysaccharide had no effect on PK15 cells’ growth at 24 h.p.i and 36 h.p.i. The results show that cell viability is not affected by Huaier polysaccharide (25, 50, 100, and 200 μg/mL). In addition, we found that Huaier polysaccharide (200 μg/mL) could not induce apoptosis in PK15 cells (Figure 1C).

### 3.2. Huaier Polysaccharide Inhibis PRV XJ5 Infection in PK15 Cells

**Figure 2:**
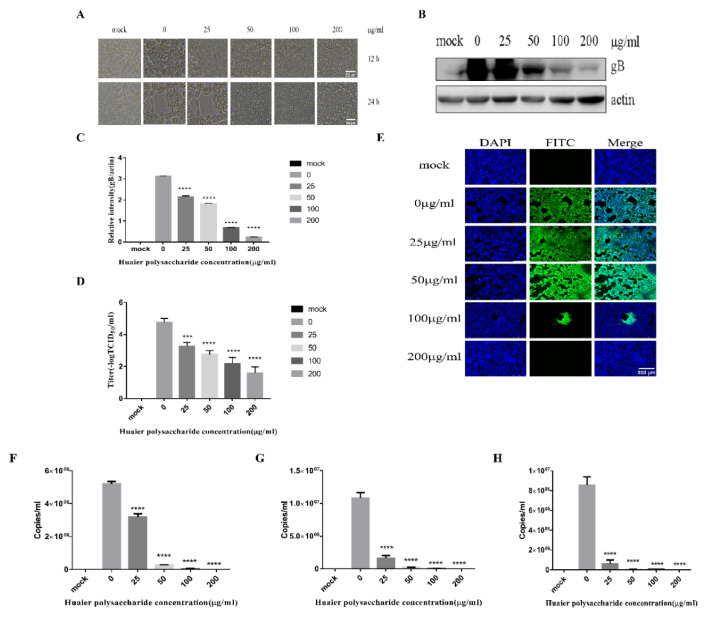
Huaier polysaccharide inhibits PRV XJ5 infection in PK15 cells. (**A**) Infected cells treated with 25, 50, 100, or 200 μg/mL Huaier polysaccharide for 12 or 24 h, showing changes in cell morphology. (**B**) gB and actin protein expression was determined through Western blot assay. (**C**) The relative intensity of intracellular gB to that of actin. Data are presented as means from three independent statistical experiments. The relative intensities of protein were quantified using Image J. Significance was analyzed using a one-tailed Student’s *t*-test. (**D**) The viral titers were evaluated through 50% tissue culture infective dose (TCID_50_), and (**E**) immunofluorescent assay (IFA) for internalized virus was performed. (**F**–**H**) PRV gB were assessed with qRT-PCR analysis in PK15 cells treated with Huaier polysaccharide (25, 50, 100, and 200 μg/mL) at 24 h.p.i. *** *p* = 0.0003, **** *p* < 0.0001. Data are shown as mean ± SD based on three independent experiments.

Figure 1 reveals that Huaier polysaccharide had no effect on PK15 cells’ growth. To investigate the role of Huaier polysaccharides in preventing PRV XJ5 infection, PK15 cells were treated as described in Section 2.3.2. PRV XJ5 (MOI = 0.1) infection resulted in an obvious cytopathic effect (CPE) in the PK15 cells (Figure 2A), which could be inhibited by Huaier polysaccharide, especially at the concentrations of 100 and 200 μg/mL. The antiviral effect of Huaier polysaccharide against PRV XJ5 was further demonstrated through Western blot analysis. gB is a protein that is essential for PRV replication [28]. Thus, we assessed the change in gB expression. The results indicated that the PRV gB protein expression level was reduced after Huaier polysaccharide treatment, with an inhibition rate of 73–94%, at 100 and 200 μg/mL concentrations (Figure 2B,C). The supernatant of the infected PK15 cells was collected to determine the viral titer in terms of the 50% tissue culture infective dose (TCID_50_). As expected, TCID_50_ analysis showed that the Huaier polysaccharide treatment reduced the production of virions; 200 μg/mL Huaier polysaccharide displayed the inhibition rate of 99.9% (Figure 2D). Furthermore, the immunofluorescent assay (IFA) confirmed that Huaier polysaccharide treatment inhibited PRV XJ5 infection in a dose-dependent manner, with the inhibition rate of 13% (25 μg/mL)–99.6% (200 μg/mL) (Figure 2E). qRT-PCR analysis indicated that Huaier polysaccharide decreased the viral DNA copies of PRV XJ5 (Figure 2F). In addition, we explored the effect of Huaier polysaccharide on PRV NT and PRV Ra by qRT-PCR. qRT-PCR analysis revealed that Huaier polysaccharide decreased the viral DNA copies of PRV NT (Figure 2G) and PRV Ra (Figure 2H). The purpose of the infective assay experiment was to emphasize that Huaier polysaccharide has a significant role in the whole process of PRV XJ5 infection of PK15 cells.

### 3.3. Huaier Polysaccharide Decreased the Adsorption and Entry of PRV XJ5

**Figure 3:**
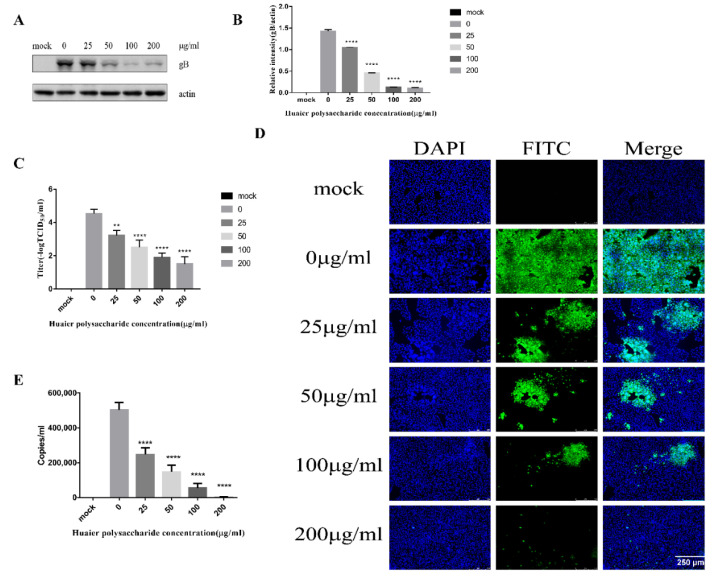
Huaier polysaccharide reduced PRV XJ5 adsorption and entry. The cells were treated according to the method described in Section 2.3.3. (**A**) Western blot showing changes in gB and actin expression. (**B**) The intensity band ratio of intracellular gB to actin. Data are presented as means from three independent statistical experiments. The intensities of protein bands were quantified using Image J. Significance was analyzed using one-tailed Student’s *t*-test. **** *p* < 0.0001. (**C**) The viral titers were evaluated through TCID_50_ assay. (**D**) The numbers of cells infected with PRV were observed through IFA. (**E**) The virus DNA copies were observed through qRT-PCR after cells were washed with citric acid and PBS to remove uninternalized virus. ** *p* = 0.003, **** *p* < 0.0001. Data are shown as mean ± SD based on three independent experiments.

The PRV life cycle comprises adsorption, entry, viral DNA replication, virion morphogenesis, and viral egress [12,26,27]. In order to further explore the role of Huaier polysaccharides in these processes of PRV XJ5 infected PK15 cells, we firstly studied the effect of Huaier polysaccharide on virus adsorption and entry. The PK15 cells were infected with PRV XJ5 (MOI = 0.1) according to the method described in Section 2.3.3. gB protein was reduced, especially in the presence of 50, 100, and 200 μg/mL Huaier polysaccharide, with inhibition rates of 51%, 75%, and 87%, respectively (Figure 3A,B). Simultaneously, the supernatant was collected, and the viral titer was determined at 24 h.p.i. The results indicated that the PRV XJ5 viral titer was decreased significantly in the cells treated with Huaier polysaccharide (Figure 3C). Furthermore, the IFA demonstrated that Huaier polysaccharide decreased the numbers of cells infected with PRV XJ5 by approximately 48–99.1% (Figure 3D). In addition, we collected cells to quantify the viral DNA copies by qRT-PCR after cells were washed with citric acid and PBS to remove uninternalized virus. qRT-PCR analysis indicated that Huaier polysaccharide decreased viral adsorption and entry in a dose-dependent manner (Figure 3E).

#### 3.3.1. Huaier Polysaccharide Attenuates PRV Adsorption

**Figure 4:**
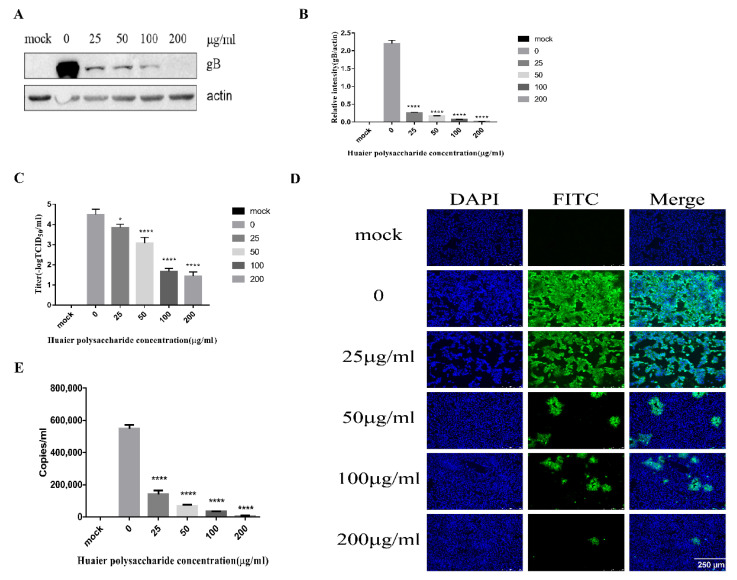
Huaier polysaccharide attenuates PRV XJ5 adsorption. PK15 cells were treated according to the method described in Section 2.3.4. (**A**) At 24 h.p.i., PRV gB and actin protein expression were analyzed through the Western blot assay. (**B**) The intensity band ratio of intracellular gB to actin. The intensities of protein bands were quantified using Image J. (**C**) The viral titers were evaluated through TCID_50_ assay. (**D**) Infected PRV XJ5 cells were observed through IFA. (**E**) The virus DNA copies were observed through qRT-PCR after cells were washed with PBS to remove unadsorbed virus. * *p* = 0.0219, **** *p* < 0.0001. Data are shown as mean ± SD based on three independent experiments.

This experiment emphasized PRV XJ5 adsorption. The adsorption of PRV occurred for 1 h at 4 °C. We performed the experiment to study virus adsorption, as described in Section 2.3.4. Figure 4A,B reveal that the Huaier polysaccharide treatment reduced PRV gB protein expression levels, with the inhibition rate of 86–99%, compared with those in untreated cells. To further assess the effect of Huaier polysaccharide on PRV XJ5 adsorption, inhibition of virus adsorption was observed through TCID_50_ assay in the cell supernatant (Figure 4C), and the IFA assay was used to observe the virus-infected cells (Figure 4D). In addition, we collected cells to quantify the viral DNA copies by qRT-PCR after cells were washed with PBS to remove unabsorbed virus. qRT-PCR analysis indicated that Huaier polysaccharide decreased viral adsorption (Figure 4E). The results indicated that Huaier polysaccharide attenuated virus adsorption by the PK15 cells.

#### 3.3.2. Huaier Polysaccharide Influences PRV Entry into PK15 Cells

**Figure 5:**
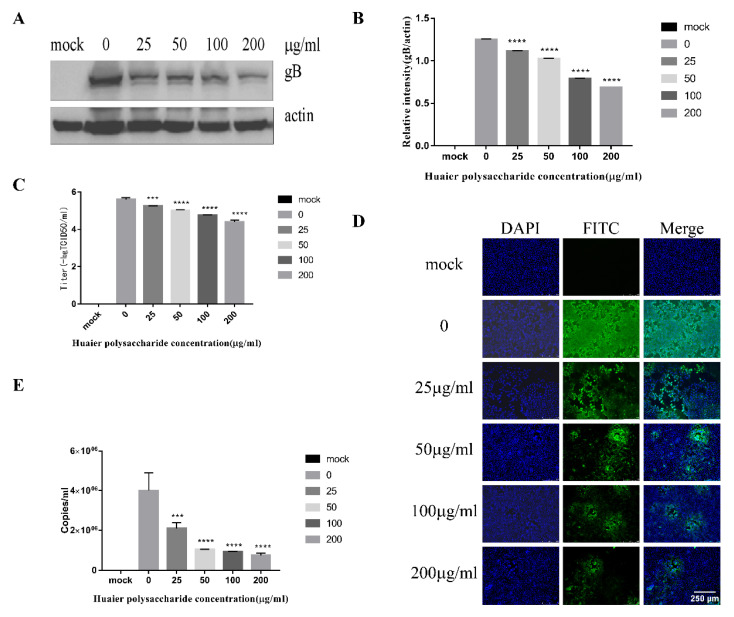
Huaier polysaccharide influences entry of pseudorabies virus in PK15 cells. The PK15 cells were treated according to the method described in Section 2.3.5. (**A**) At 24 h.p.i, PRV gB and actin protein expression were quantified through Western blot assay. (**B**) The relative intensity of intracellular gB to that of actin. Data are presented as the mean value from three independent statistical experiments. The relative intensities of protein were quantified using Image J. Significance was analyzed using the one-tailed Student’s *t*-test. (**C**) The viral titers were evaluated through TCID_50_ assay. (**D**) Immunofluorescence assay (IFA) for internalized virus was performed. (**E**) Intracellular viral DNA copies were analyzed by qRT-PCR after cells were washed with citric acid and PBS to remove uninternalized virus. *** *p* = 0.0001, **** *p* < 0.0001. Data are shown as mean ± SD based on three independent experiments.

This experiment explored separately the entry of PRV at 1 h and 37 °C. We performed the experiment to study PRV entry according to the method described in Section 2.3.5. Western blot analysis showed that Huaier polysaccharide decreased the expression of PRV gB, with the PRV entry inhibition rate being 13–47% (Figure 5A,B). The TCID_50_ assay revealed that the PRV XJ5 titers were decreased (Figure 5C). The cells infected with PRV were examined through IFA (Figure 5D). In addition, we collected cells to quantify the viral DNA copies by qRT-PCR after cells were washed with citric acid and PBS to remove uninternalized virus. qRT-PCR analysis indicated that Huaier polysaccharide decreased viral entry in a dose-dependent manner (Figure 5E). The results confirmed that Huaier polysaccharide influences PRV XJ5 entry. However, the inhibition rate of PRV entry by Huaier polysaccharide was lower than the observed inhibition rate of PRV adsorption. The results indicate that Huaier polysaccharide might have a prominent role in PRV adsorption. 

### 3.4. Huaier Polysaccharide Mildly Reduces PRV XJ5 Replication in PK15 Cells

**Figure 6:**
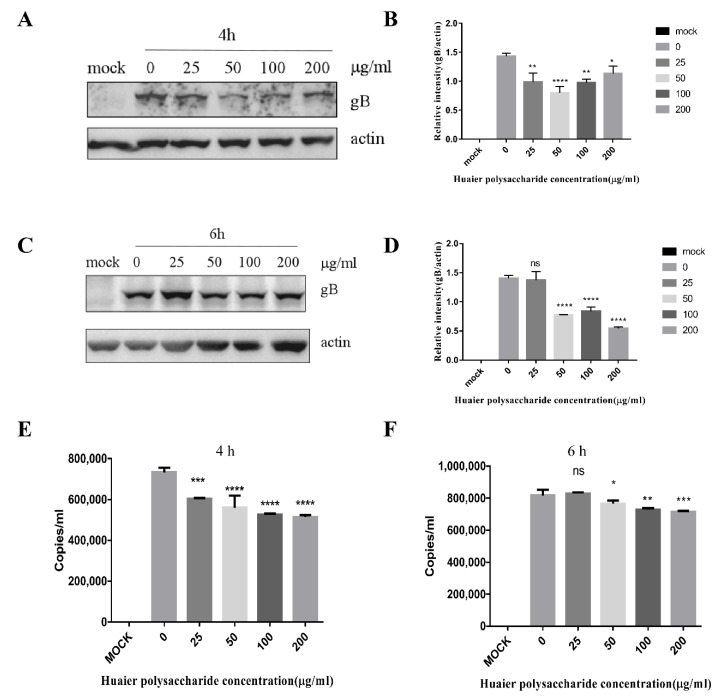
Huaier polysaccharide affects PRV XJ5 replication in PK15 cells. The PK15 cells were treated according to the method described in Section 2.3.6. At 4 and 6 h.p.i., the cells were collected to evaluate the expression of PRV gB and actin proteins through Western blotting (**A**,**C**). (**B**,**D**) The relative intensity of intracellular gB to that of actin. The relative intensities of protein were quantified using Image J. (**E**,**F**) PRV viral DNA copies were analyzed by qRT-PCR at 4 and 6 h.p.i. Significance was analyzed using the one-tailed Student’s *t*-test. NS, * *p* = 0.0299, ** *p* = 0.0017, *** *p* = 0.0001, **** *p* < 0.0001. Data are shown as mean ± SD based on three independent experiments.

The adsorption of PRV requires 1 h and occurs at 4 °C; entry of PRV requires 1 h and occurs at 37 °C [29]. The life cycle of PRV is completed in approximately 6 h. Hence, we chose the time periods of 4 and 6 h to study PRV replication. The expression of virus protein gB and actin was analyzed through the Western blot assay at 4 and 6 h.p.i. The DNA copies were analyzed by qRT-PCR. The results indicated that Huaier polysaccharide has a slight effect on gB expression (Figure 6A–F), indicating that Huaier polysaccharide mildly affects virus replication.

### 3.5. Huaier Polysaccharide May Directly Inactivate PRV XJ5

**Figure 7:**
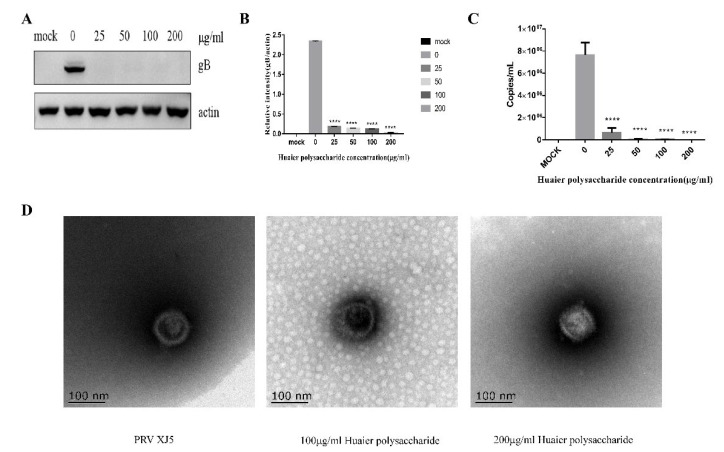
Huaier polysaccharide may directly inactivate PRV XJ5. PRV XJ5 (MOI = 0.1) was treated according to the method described in Section 2.3.8. (**A**) PRV gB was analyzed through Western blotting. (**B**) The relative intensity of intracellular gB to that of actin. (**C**) PRV viral DNA copies were analyzed by qRT-PCR at 24 h.p.i (**D**) PRV particle morphology was observed by electron microscopy. The relative intensities of protein were quantified using Image J. Significance was analyzed using the one-tailed Student’s *t*-test. **** *p* < 0.0001. Data are shown as mean ± SD based on three independent experiments.

PRV XJ5 (MOI = 0.1) was pretreated with different concentrations of Huaier polysaccharide for 1 h at 37 °C, and the PK15 cells were infected for 24 h. The cells were collected to assess PRV gB expression through Western blotting. Figure 7A,B illustrate that Huaier polysaccharide reduced PRV gB expression. Figure 7C indicated that virus copies were reduced significantly. In addition, the transmission electron microscope experiment revealed that the PRV envelope was destroyed by Huaier polysaccharide (Figure 7D). These results indicate that Huaier polysaccharide may directly inactivate PRV XJ5.

## 4. Discussion

Huaier has been used as a traditional Chinese herbal medicine for the treatment of various diseases for thousands of years. In clinical treatment, Huaier polysaccharide has demonstrated a promising auxiliary effect in the treatment of breast cancer, liver cancer, and gastric cancer [30]. Increasing evidence suggests that Huaier polysaccharide can inhibit cell proliferation [31,32], cause cell cycle arrest [33,34], and induce apoptosis [35,36] in cancer cells. In a study, the bi-directional solid fermentation product extract of Huaier with Radix Isatidis (TIF) could upregulate the expression of p53 and caspase-3 in both SK-BR-3 and MDA-MB-231 cell lines [37]. In another study, researchers found that Huaier polysaccharide could inhibit proliferation and promote apoptosis by increasing miR-26b-5p expression in pulmonary cancer cells [38]. Additionally, Huaier polysaccharide extract was found to impair genes related to cell division, the cell cycle, cell cycle phases, and DNA repair [39]. Moreover, in vitro assays confirmed that Huaier polysaccharide could markedly increase the persistence of γ-H2A.X foci and interfere with the homologous recombination pathway [39]. 

The present study focused on determining the effect of Huaier polysaccharide on virus infection and confirmed that Huaier polysaccharide can affect multiple life cycle stages of PRV XJ5 virus to exert its antiviral effects. PK15 cells are generally used in in vitro PRV research [40]. Huaier polysaccharide mainly affects the adsorption of PRV XJ5 on PK15 cells, and the effect is dose-dependent. The results of qRT-PCR further illustrated this phenomenon. In a study by Zhang et al., the number of successfully invading/migrating cells and the wound closure rate established through the transwell assay and scratch assay, respectively, were reported to be decreased significantly by Huaier polysaccharide, which proved the anti-metastasis effect of Huaier polysaccharide [20]. Thus, we speculate that Huaier polysaccharide inhibits PRV adsorption through its anti-metastasis action; however, the confirmation of this assumption warrants further study. In addition, we revealed that Huaier polysaccharide inhibited PRV entry into PK15 cells by qRT-PCR. Moreover, we found that Huaier polysaccharide prevents virus infection, possibly by binding to a certain receptor on the cell surface, and it protects the cells probably by directly inactivating the virus. The polysaccharide might exert a weak effect on PRV XJ5 replication.

Studies have been focusing on one of the main pathways of programmed cell death after viral infection [41]. The host cell can destroy virus-infected cells through apoptosis, thereby preventing virus infection [42]. Huaier polysaccharide plays a central role in activating the caspase-3 signaling pathway for apoptosis induction [43]. The Huaier polysaccharide component SP1 (a type of purified Huaier polysaccharide) could increase the proportion of Bax/Bcl-2 through the MTDH signaling pathway, which could be another potential mechanism of Huaier in apoptosis induction in breast cancer cells [22]. Moreover, 18β-glycyrrhetinic acid inhibits apoptosis of the cells infected with rotavirus SA11 to decrease rotavirus SA11 infection by Fas (CD95) and FasL (CD178). Similarly, Huaier polysaccharide may depend on Fas (CD95) and FasL (CD178) to regulate apoptosis to inhibit PRV infection [44]. We aim to investigate this assumption in our future studies. 

## 5. Conclusions

In this study, we revealed that Huaier polysaccharide acts against PRV in PK15 cells by blocking PRV adsorption and entry. Hence, Huaier polysaccharide could be further developed as an antiviral agent against PRV infection. Although some studies have investigated the molecular mechanisms, pharmacokinetic and pharmacodynamic models should be developed for an in-depth understanding of the mechanism.

## References

[1] Lee J.Y., Wilson M.R. (1979). A review of pseudorabies (Aujeszky’s disease) in pigs. Can. Vet. J..

[2] Xia L.M., Sun Q.Y., Wang J.J., Chen Q., Liu P.H., Shen C.J., Sun J.H., Tu Y.P., Shen S.F., Zhu J.C. (2018). Epidemiology of pseudorabies in intensive pig farms in Shanghai, China: Herd-level prevalence and risk factors. Prev. Vet. Med..

[3] Sun Y., Luo Y.Z., Wang C.-H., Yuan J., Li N., Song K., Qiu H.-J. (2016). Control of swine pseudorabies in China: Opportunities and limitations. Vet. Microbiol..

[4] Klupp B.G. (2021). Pseudorabies Virus Infections. Pathogens.

[5] Minamiguchi K., Kojima S., Sakumoto K., Kirisawa R. (2019). Isolation and molecular characterization of a variant of Chinese gC-genotype II pseudorabies virus from a hunting dog infected by biting a wild boar in Japan and its pathogenicity in a mouse model. Virus Genes.

[6] Cheng Z., Kong Z., Liu P., Fu Z., Zhang J., Liu M., Shang Y. (2020). Natural infection of a variant pseudorabies virus leads to bovine death in China. Transbound. Emerg. Dis..

[7] Kong H., Zhang K., Liu Y., Shang Y., Wu B., Liu X. (2013). Attenuated live vaccine (Bartha-K16) caused pseudorabies (Aujeszky’s disease) in sheep. Vet. Res. Commun..

[8] Kaneko C., Kaneko Y., Sudaryatma P.E., Mekata H., Kirino Y., Yamaguchi R., Okabayashi T. (2021). Pseudorabies virus infection in hunting dogs in Oita, Japan: Report from a prefecture free from Aujeszky’s disease in domestic pigs. J. Vet. Med. Sci..

[9] Jin H.-L., Gao S.-M., Liu Y., Zhang S.-F., Hu R.-L. (2016). Pseudorabies in farmed foxes fed pig offal in Shandong province, China. Arch. Virol..

[10] Freuling C.M., Mueller T.F., Mettenleiter T.C. (2017). Vaccines against pseudorabies virus (PrV). Vet. Microbiol..

[11] Mettenleiter T.C. (2000). Aujeszky’s disease (pseudorabies) virus: The virus and molecular pathogenesis—State of the art, June 1999. Vet. Res..

[12] Tan L., Yao J., Yang Y., Luo W., Yuan X., Yang L., Wang A. (2021). Current Status and Challenge of Pseudorabies Virus Infection in China. Virol. Sin..

[13] Szpara M.L., Tafuri Y.R., Parsons L., Shamim S.R., Verstrepen K.J., Legendre M., Enquist L.W. (2011). A Wide Extent of Inter-Strain Diversity in Virulent and Vaccine Strains of Alphaherpesviruses. PLoS Pathog..

[14] Ye C., Guo J.-C., Gao J.-C., Wang T.-Y., Zhao K., Chang X.-B., Wang Q., Peng J.-M., Tian Z.-J., Cai X.-H. (2016). Genomic analyses reveal that partial sequence of an earlier pseudorabies virus in China is originated from a Bartha-vaccine-like strain. Virology.

[15] Ai J.-W., Weng S.-S., Cheng Q., Cui P., Li Y.-J., Wu H.-L., Zhu Y.-M., Xu B., Zhang W.-H. (2018). Human Endophthalmitis Caused by Pseudorabies Virus Infection, China, 2017. Emerg. Infect. Dis..

[16] Liu Q., Wang X., Xie C., Ding S., Yang H., Guo S., Li J., Qin L., Ban F., Wang D. (2020). A novel human acute encephalitis caused by pseudorabies virus variant strain. Clin. Infect. Dis..

[17] Yang X., Guan H., Li C., Li Y., Wang S., Zhao X., Zhao Y., Liu Y. (2019). Characteristics of human encephalitis caused by pseudorabies virus: A case series study. Int. J. Infect. Dis..

[18] Liu S.-H., Chuang W.-C., Lam W., Jiang Z., Cheng Y.-C. (2015). Safety Surveillance of Traditional Chinese Medicine: Current and Future. Drug Saf..

[19] Kannan S., Kolandaivel P. (2017). Antiviral potential of natural compounds against influenza virus hemagglutinin. Comput. Biol. Chem..

[20] Zhang N., Kong X., Yan S., Yuan C., Yang Q. (2010). Huaier aqueous extract inhibits proliferation of breast cancer cells by inducing apoptosis. Cancer Sci..

[21] Kong X., Ding X., Yang Q. (2015). Identification of multi-target effects of Huaier aqueous extract via microarray profiling in triple-negative breast cancer cells. Int. J. Oncol..

[22] Luo Z., Hu X., Xiong H., Qiu H., Yuan X., Zhu F., Wang Y., Zou Y. (2016). A polysaccharide from Huaier induced apoptosis in MCF-7 breast cancer cells via down-regulation of MTDH protein. Carbohydr. Polym..

[23] Chen Q., Shu C., Laurence A.D., Chen Y., Peng B.G., Zhen Z.J., Cai J.Q., Ding Y.T., Li L.Q., Zhang Y.B. (2018). Effect of Huaier granule on recurrence after curative resection of HCC: A multicentre, randomised clinical trial. Gut.

[24] Yao X., Wu W., Qu K., Xi W. (2020). Traditional Chinese biomedical preparation (Huaier Granule) for breast cancer: A PRISMA-compliant meta-analysis. Biosci. Rep..

[25] Wang M., Hu Y., Hou L., Pan Q., Tang P., Jiang J. (2019). A clinical study on the use of Huaier granules in post-surgical treatment of triple-negative breast cancer. Gland Surg..

[26] Enquist L.W., Husak P.J., Banfield B.W., Smith G.A. (1998). Infection and spread of alphaherpesviruses in the nervous system. Adv. Virus Res..

[27] Mettenleiter T.C. (2004). Budding events in herpesvirus morphogenesis. Virus Res..

[28] Ren J., Wang H., Zhou L., Ge X., Guo X., Han J., Yang H. (2020). Glycoproteins C and D of PRV Strain HB1201 Contribute Individually to the Escape From Bartha-K61 Vaccine-Induced Immunity. Front. Microbiol..

[29] He W., Zhai X., Su J., Ye R., Zheng Y., Su S. (2019). Antiviral Activity of Germacrone against Pseudorabies Virus in Vitro. Pathogens.

[30] Qi J., Xie F.-j., Liu S., Yao C.-y., Liu W.-h., Cai G.-q., Liao G.-q. (2020). Huaier Granule Combined with Tegafur Gimeracil Oteracil Potassium Promotes Stage IIb Gastric Cancer Prognosis and Induces Gastric Cancer Cell Apoptosis by Regulating Livin. Biomed. Res. Int..

[31] Yang A., Zhao Y., Wang Y., Zha X., Zhao Y., Tu P., Hu Z. (2018). Huaier suppresses proliferative and metastatic potential of prostate cancer PC3 cells via downregulation of Lamin B1 and induction of autophagy. Oncol. Rep..

[32] Wang Y., Lv H., Xu Z., Sun J., Ni Y., Chen Z., Cheng X. (2019). Huaier n-butanol extract suppresses proliferation and metastasis of gastric cancer via c-Myc-Bmi1 axis. Sci. Rep..

[33] Yan L., Liu X., Yin A., Wei Y., Yang Q., Kong B. (2015). Huaier aqueous extract inhibits cervical cancer cell proliferation via JNK/p38 pathway. Int. J. Oncol..

[34] Hu Z., Yang A., Su G., Zhao Y., Wang Y., Chai X., Tu P. (2016). Huaier restrains proliferative and invasive potential of human hepatoma SKHEP-1 cells partially through decreased Lamin B1 and elevated NOV. Sci. Rep..

[35] Xie H.-X., Xu Z.-Y., Tang J.-N., Du Y.-A., Huang L., Yu P.-F., Cheng X.-D. (2015). Effect of Huaier on the proliferation and apoptosis of human gastric cancer cells through modulation of the PI3K/AKT signaling pathway. Exp. Ther. Med..

[36] Heller M.J. (2002). DNA microarray technology: Devices, systems, and applications. Annu. Rev. Biomed. Eng..

[37] Liu Z., Tang Y., Zhou R., Shi X., Zhang H., Liu T., Lian Z., Shi X. (2018). Bi-directional solid fermentation products of Trametes robiniophila Murr with Radix Isatidis inhibit proliferation and metastasis of breast cancer cells. J. Chin. Med. Assoc..

[38] Wu T., Chen W., Liu S., Lu H., Wang H., Kong D., Huang X., Kong Q., Ning Y., Lu Z. (2014). Huaier suppresses proliferation and induces apoptosis in human pulmonary cancer cells via upregulation of miR-26b-5p. Febs Lett..

[39] Ding X., Yang Q., Kong X., Haffty B.G., Gao S., Moran M.S. (2016). Radiosensitization effect of Huaier on breast cancer cells. Oncol. Rep..

[40] Yu T., Chen F., Ku X., Fan J., Zhu Y., Ma H., Li S., Wu B., He Q. (2016). Growth characteristics and complete genomic sequence analysis of a novel pseudorabies virus in China. Virus Genes.

[41] Danthi P. (2016). Viruses and the Diversity of Cell Death. Annu. Rev. Virol..

[42] Zhou X., Jiang W., Liu Z., Liu S., Liang X. (2017). Virus Infection and Death Receptor-Mediated Apoptosis. Viruses.

[43] Porter A.G., Jänicke R.U. (1999). Emerging roles of caspase-3 in apoptosis. Cell Death Differ..

[44] Wang X., Xie F., Zhou X., Chen T., Xue Y., Wang W. (2021). 18β-Glycyrrhetinic acid inhibits the apoptosis of cells infected with rotavirus SA11 via the Fas/FasL pathway. Pharm. Biol..

